# Case Report: A rare hyperplastic polyp with synchronous high-grade intraepithelial neoplasia in both glandular and squamous epithelium in autoimmune gastritis

**DOI:** 10.3389/fonc.2025.1641188

**Published:** 2025-08-15

**Authors:** Dian Zhang, Xueqin Chen, Maher Hendi, Wen Tang, Chenfei Tan, Juan Liu, Weiling Hu

**Affiliations:** ^1^ Department of Gastroenterology, Sir Run Run Shaw Hospital, Medical School, Zhejiang University, Hangzhou, China; ^2^ School of Medicine, Shaoxing University, Shaoxing, China; ^3^ Department of Surgery, Sir Run Run Shaw Hospital, Medical School, Zhejiang University, Hangzhou, China; ^4^ Department of Pathology, Sir Run Run Shaw Hospital, Medical School, Zhejiang University, Hangzhou, China; ^5^ School of Medicine, Zhejiang University, Hangzhou, China

**Keywords:** autoimmune gastritis, hyperplastic polyp, squamous epithelium, high-grade intraepithelial neoplasia, precancerous condition

## Abstract

A 68-year-old man came to evaluate a gastric polyp discovered during a routine gastroscopy. After endoscopic mucosal resection, pathological findings confirmed that it’s a hyperplastic polyp. Notably, squamous metaplasia was observed within the hyperplastic polyp, and both squamous and glandular epithelium exhibited high-grade intraepithelial neoplasia. Besides, the diagnosis of autoimmune gastritis was established by comprehensive assessment including gastric endoscopic findings, histopathological examination and serological studies. The patient experienced no postoperative discomfort and had oral medication for two weeks. In this paper, we presents a first case worldwide of hyperplastic polyp with synchronous high-grade intraepithelial neoplasia in both glandular and squamous epithelium in autoimmune gastritist. In previous cases, the occurrence of hyperplastic polyps with neoplastic transformations or squamous metaplasia is very rare, but what we found this time was the concurrance of both lesions on hyperplastic polyps. This extremely rare case not only provides further clinical evidence for the metaplasia and neoplastic transformation potential of hyperplastic polyps, but also highlights the necessity of regular follow-up examination for autoimmune gastritis - a well-established precancerous condition.

## 1 Introduction

Autoimmune gastritis (AIG), a chronic inflammatory disease, is characterized by gastric mucosa atrophy, parietal cell destruction and intrinsic factor loss (1). The basic lesions of AIG include neuroendocrine cell hyperplasia, pyloric gland adenoma, neuroendocrine tumors (2), etc. Due to its nonspecific clinical presentation, the diagnosis is mainly relied on endoscopy, histopathology and serological studies (1). Endoscopically, autoimmune gastritis typically manifests as corpus-dominant mucosal atrophy (3). Meanwhile, hyperplastic polyps (HPs) can sometimes be observed in the stomach (2). Gastric yperplastic polyps (GHPs), predominantly located in the gastric antrum, represent benign epithelial lesions resulting from expansion and exfoliation of gastric foveolar cells (4, 5). Although long-term autoimmune gastritis is considered as precancerous disease, metaplasia or malignant transformation of hyperplastic polyps remains exceedingly rare (6).

Here, we present a rare case of gastric hyperplastic polyp with synchronous high-grade intraepithelial neoplasia in both glandular and squamous epithelium in autoimmune gastritis. The lesion was accidentally discovered during a routine endoscopy. To our knowledge, this is the first reported case of its kind worldwide.

## 2 Case description

The patient, a 68-year-old male, presented for medical consultation asymptomatically after a gastric polyp was discovered during a health check-up.

**Figure 1:**
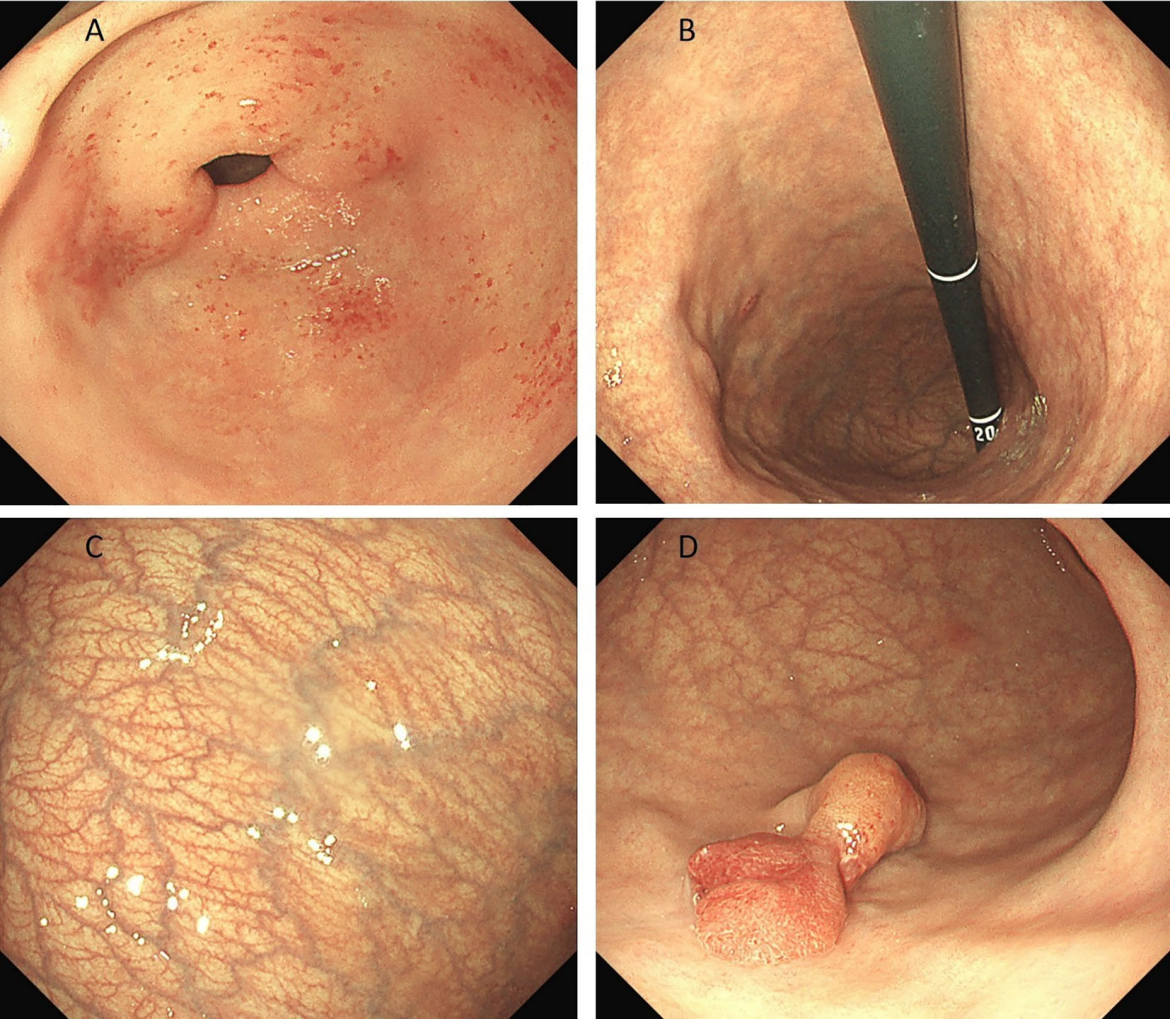
**(A-C)** shows significant atrophy of the gastric body, thinning of the gastric wall, no mucosal folds and a visible vascular network. Atrophy of the antrum was not obvious. **(D)** shows a broad polyp measuring approximately 1.8 cm in size in the lower part of the gastric body, with a noticeably congested and rough surface.

**Figure 2:**
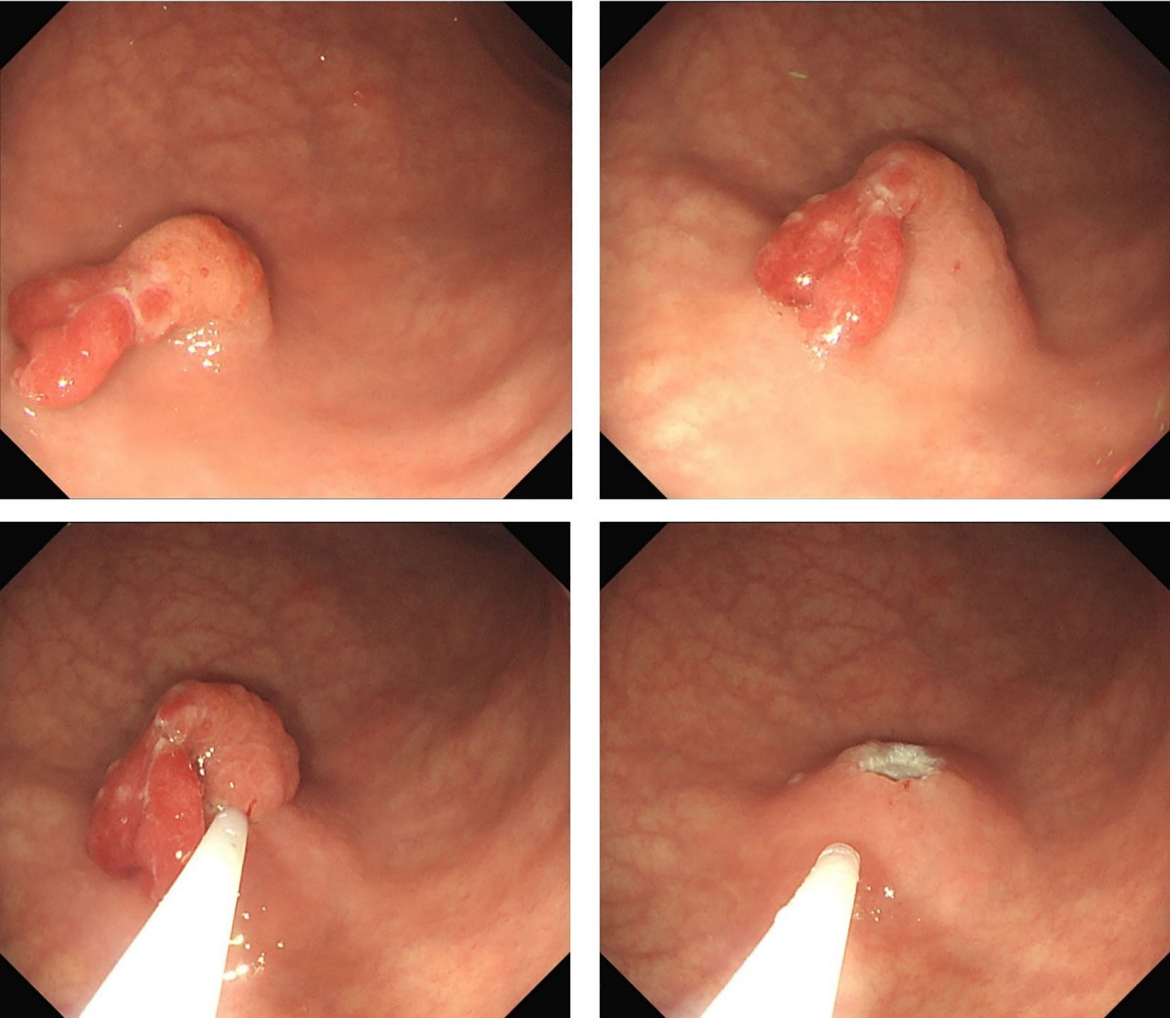
Shows the procedure of endoscopic mucosal resection (EMR).

Preoperative endoscopy revealed significant atrophy of the gastric body, thinning of the gastric wall, that the mucosal folds were no longer visible after inflation, and that the vascular network was visible. Atrophy of the antrum was not obvious. On the basis of the above endoscopic findings, autoimmune gastritis was suspected (**Figures 1A–C**). Preoperative pathology suggested a hyperplastic polyp, and the patient was scheduled for endoscopic polypectomy. During endoscopic polyp resection, we identified a broad-based polyp approximately 1.8 cm in size in the lower part of the gastric body, with a noticeably congested and rough surface (**Figure 1D**). We performed endoscopic mucosal resection (EMR) (**Figure 2**). The polyp was sent for pathological examination.

**Figure 3:**
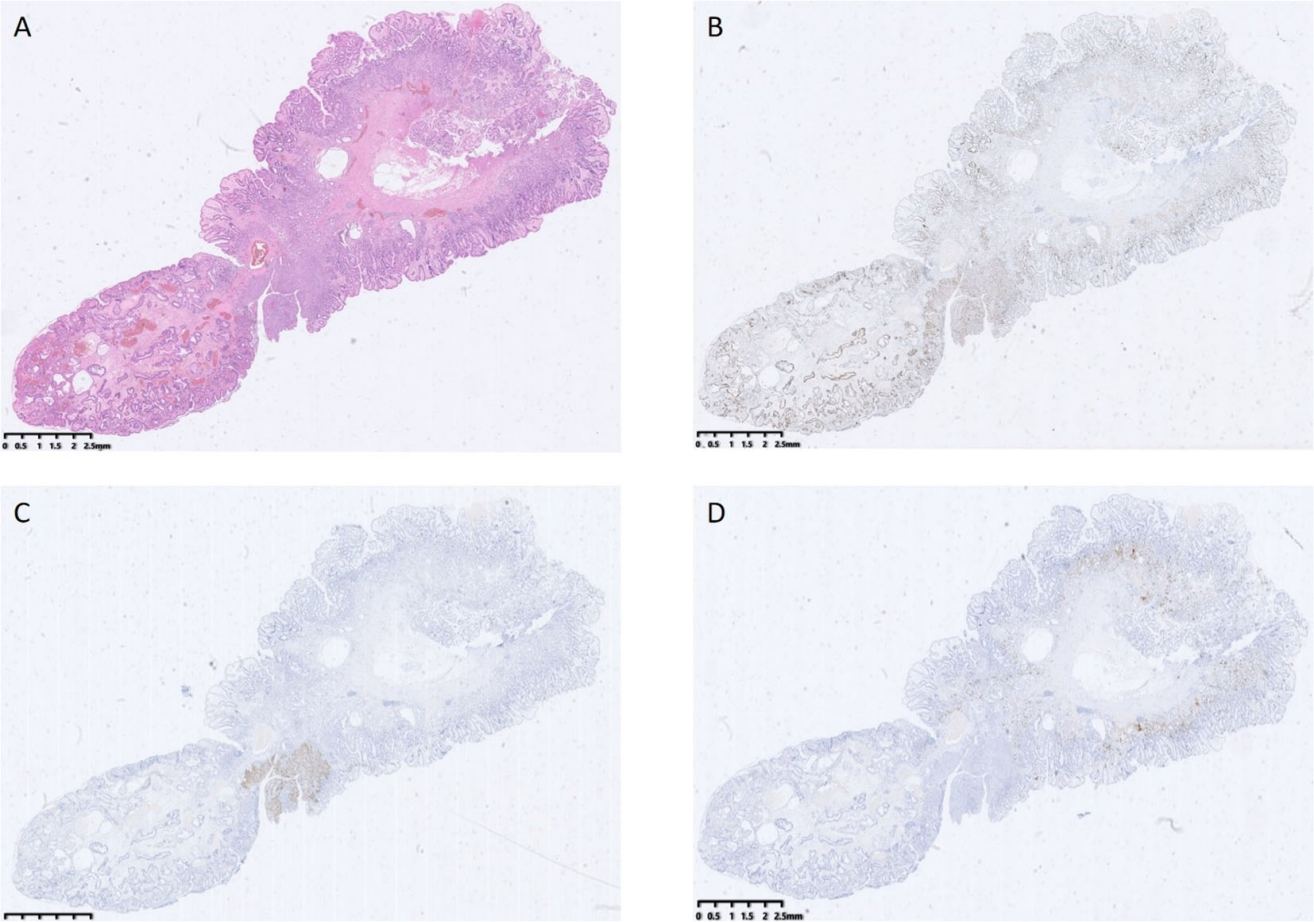
**(A)** shows a hyperplastic polyp with high-grade intraepithelial neoplasia of the glandular epithelium, along with squamous metaplasia and high-grade intraepithelial neoplasia. **(B)** shows the abnormal expression and distribution of Ki67. **(C)** shows local positive expression of P63. **(D)** shows positive expression of CgA.

**Figure 4:**
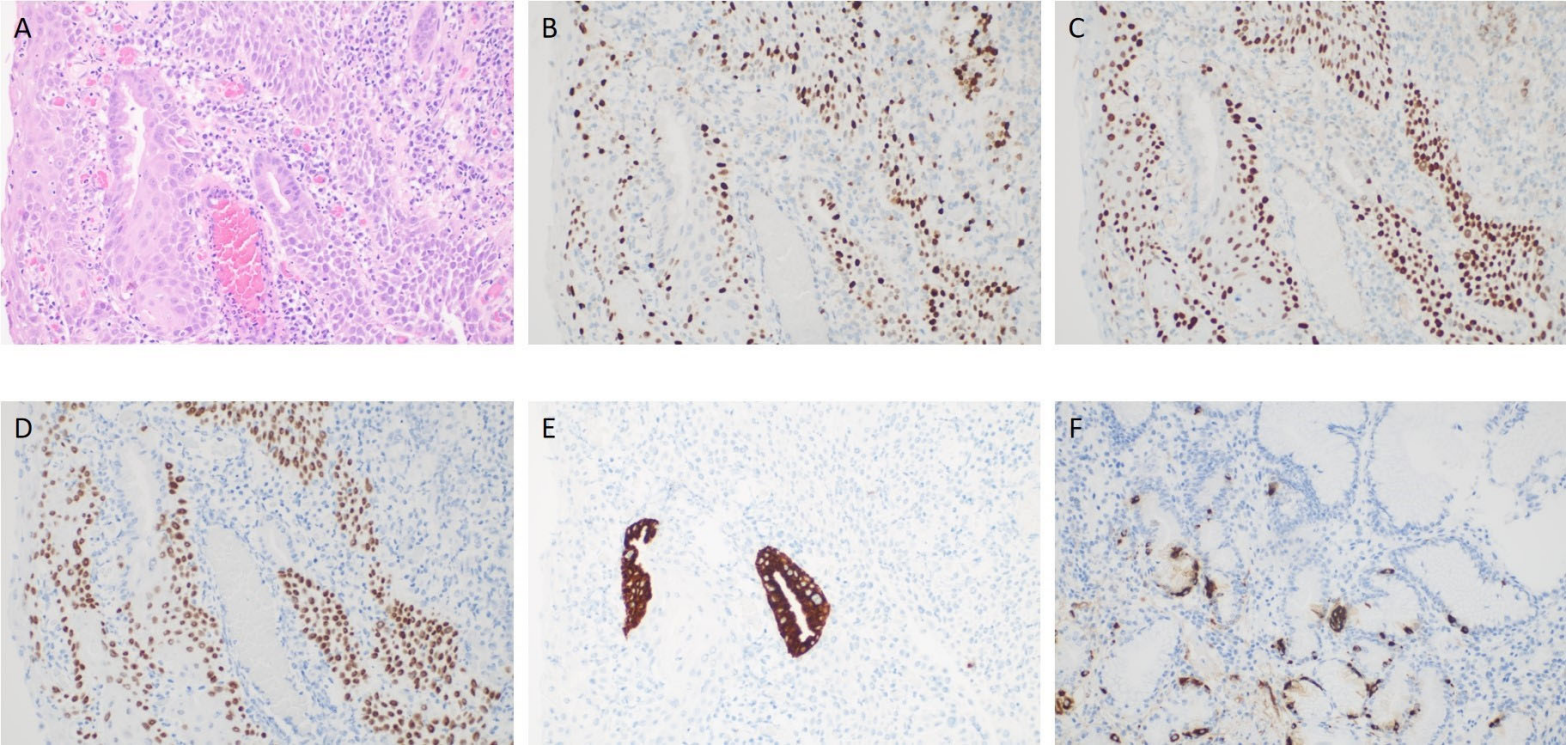
**(A)** shows high-grade intraepithelial neoplasia of the glandular epithelium, along with squamous metaplasia and high-grade intraepithelial neoplasia. **(B)** shows the abnormal expression and distribution of Ki67. **(C)** shows positive expression of P53. **(D)** shows positive expression of P63. **(E)** shows positive expression of MUC5. **(F)** shows positive expression of CgA.

Post-endoscopy pathology revealed a hyperplastic polyp with high-grade intraepithelial neoplasia of the glandular epithelium, along with squamous metaplasia with high-grade intraepithelial neoplasia, which is very rare in the stomach (**Figures 3**, **4A–E**). Histopathological analysis demonstrated that squamous metaplasia with malignant transformation is evident, and immunohistochemical staining shows positivity for P53. All the lesions were mucosal-confined without submucosal invasion. The base resection margins were negative. The surrounding gastric body mucosa showed atrophy, pyloric gland metaplasia, and microscopic nodular hyperplasia of neuroendocrine cells (**Figures 3D**, **4F**). The diagnosis of autoimmune gastritis was also proved.

The laboratory examination revealed that pepsinogen I level had significantly decreased to 4.43 ng/mL and that his gastrin level had markedly increased to 84.1 pmol/ml. Importantly, the patient’s serum anti-parietal cell antibody (PCA) IgG level and anti-intrinsic factor antibody (IFA) IgG level elevated to 52.16 RU/ml and 47.91 RU/ml, respectively. These findings further confirmed the diagnosis of autoimmune gastritis.

The patient did not experience any discomfort after polyp removal and was treated with oral medication for two weeks after undergoing EMR. The patient is recommended to undergo a 3-month gastroscopy.

## 3 Discussion

Recent years have witnessed a rising prevalence of autoimmune gastritis. Characteristic endoscopic hallmarks pallor and thinning of gastric mucosal, visibility of the vascular network, and loss of gastric fold (5). As a precancerous disease of the stomach (4), autoimmune gastritis is the first progress of a multistep precancerous cascade (7).

According to statistics, hyperplastic polyps represent approximately 30-93% of gastric polyps (4). Abnormal mucosa can usually generate hyperplastic polyps, especially in autoimmune gastritis (8), which is one of the main pathologic sources of GHPs (5). Autoimmune gastritis and related diseases often trigger chronic reactive or active inflammation of mucosa, promoting pit cells expansion and subsequent polyp formation (9). Autoimmune gastritis causes atrophy of secretory acid, leading to pronounced hypergastrinemia (10, 11). Hypergastrinemia is a carcinogenic factor which may induce the development of hyperplastic polyps and adenomas. Additionally, post-injury regeneration and proliferation of mucosa contribute to polyp development. Although exact mechanism is unclear, local changes or cell mutations of origin can affect polyp growth regulation (10).

Gastric hyperplastic polyps have an extremely low probability of malignant transformation (12). Current literature reports dysplasia and malignant transformation of GHPs rates ranging from 1.9% to 19% and 0.6% to 2.1%,respectively (13). For hyperplastic polyps, actually, size larger than 1 cm are recognized as a risk factor of neoplastic transformation. Larger GHPs, higher cancerous change risk. Because persistent growth of GHPs requires sustained exposure to stimulating factors (4). In our case, 1.8 cm polyp size obviously increased the malignancy risk. Yao et al. summarized four predictors of malignancy in gastric hyperplastic polyp: well-differentiated histology, dysplasia, mucin phenotype belonging to the gastric type, and expression of P53 (14). Molecular analysis indicates that experission of p53, K-ras, microsatellite instability, p21WAF1/CIP1 and cyclin D1 might be meaningful in transformation process (15). Certain membrane proteins called claudins, which are located at intracellular junctions, are responsible for cell membrane integrity and appear to be markers of gastric hyperplastic polyp malignant transformation (14). There are evidences suggested that yperplasia-dysplasia-carcinoma sequence is the development path of neoplastic transformation (16).

Metaplasia refers to the replacement of one differentiated cell type by another type in the same tissue. The specific metaplasia type vary by tissue origin, which is broadly classified as squamous metaplasia, intestinal metaplasia and acina-ductal metaplasia. Continued exposure to metaplasia-promoting factors may induce metaplasia advance to dysplasia and even malignancy (17). Gastrointestinal metaplasia is associated with chronic gastritis and mucosal atrophy (18). In our case, the chronic inflammation and mucosal damage induced by autoimmune gastritis may explain the occurrence of squamous metaplasia within the hyperplastic polyp. However, intestinal metaplasia remains the most common gastric metaplasia (17), whereas the squamous epithelium is quite rare (19). The underlying mechanism of gastric squamous metaplasia is still unknow. Research proposes three possible origins: squamous differentiation within pre-existing adenocarcinoma, a metaplastic squamous epithelium or nests of ectopic squamous epithelium, and undifferentiated pluripotent stem cells (18). These assumptions are supported by prior case reports documenting gastric squamous cells in patients with peptic ulcers, syphilis, gastric tuberculosis, and pernicious anemia (20, 21). Another theory suggests that squamation may arise from altered differentiation of gastric stem cells during regeneration (22). Although gastric squamous metaplasia is typically benign, rare cases may progress to squamous cell carcinoma (23).

Generally, both malignant transformation and squamous metaplasia in gastric hyperplastic polyps exhibit exceptionally low incidence rates. The coexistence of squamous metaplasia and malignant alternation is even rarer, with only a handful of cases reported in English journals to date (19). Notably, these previously reported lesions exclusively involved gastric mucosa rather than polyps.

To our knowledge, this case represents the first documented case of malignant transformation in a hyperplastic polyp exhibiting squamous differentiation. The case is extremely rare in that long-term gastric mucosal damage caused by autoimmune gastritis led to the development and subsequent malignant transformation of gastric hyperplastic polyps with a squamous morphology; notably, some squamous polyps may become cancerous.

However, there are still some shortcomings in our case report. Firstly, the preoperative biopsy identified the polyp as hyperplastic, leading to routine gastroscopy and resection without magnification. Consequently, detailed surface structure analysis was unavailable—a significant limitation. Secondly, the patient’s refusal of gastroscopy reexamination during follow-up left us unaware of the gastritis status or polyp recurrence. So we should pay enough attention to atrophic gastritis patients. These findings underscore the importance of routine magnifying endoscopy for autoimmune gastritis patients to strive for early diagnosis and treatment.

In future clinical practice, preliminary assessment of polyp nature under gastroscopy is a meaningful topic. We can proceed in the following directions: 1) Establishment of standardized magnifying endoscopy screening protocols for autoimmune gastritis patients; 2)Development of a comprehensive polyp image database for dysplastic feature analysis; 3) Creation of clinical data-driven predictive models for assessing polyp malignant transformation risks; 4) Investigation of AI-assisted diagnostic systems for early lesion detection.

## 4 Conclusion

In summary, this case report a highly unusual hyperplastic polyp arising in autoimmune gastritis. It highlights the importance of maintaining a high index of suspicion for autoimmune gastritis during endoscopic examinations. Relevant serological testing should be performed in patients with compatible histopathological findings to confirm the diagnosis of this precancerous condition. What’s more, doctors must recognize the oncogenic potential of hyperplastic polyps, particularly their propensity for malignant transformation and squamous metaplasia, which may serve as critical transitional stages in gastric cancer. Clinicians should make comprehensive assessment, avoiding overly simplistic therapeutic approaches. Early assessment and intervention are crucial to gastric cancer prevention.

## Data Availability

The original contributions presented in the study are included in the article/supplementary material. Further inquiries can be directed to the corresponding author.
